# Allosteric modulation of Ras and the PI3K/AKT/mTOR pathway: emerging therapeutic opportunities

**DOI:** 10.3389/fphys.2014.00478

**Published:** 2014-12-16

**Authors:** Paul A. Hubbard, Colleen L. Moody, Ramachandran Murali

**Affiliations:** ^1^Department of Biomedical Sciences, Cedars-Sinai Medical CenterLos Angeles, CA, USA; ^2^Department of Pathology and Laboratory Medicine, University of PennsylvaniaPhiladelphia, PA, USA

**Keywords:** allostery, kinase inhibitor, K-Ras, PI3K/AKT/mTOR pathway, drug design, pancreatic cancer

## Abstract

GTPases and kinases are two predominant signaling modules that regulate cell fate. Dysregulation of Ras, a GTPase, and the three eponymous kinases that form key nodes of the associated phosphatidylinositol 4,5-bisphosphate 3-kinase (PI3K)/AKT/mTOR pathway have been implicated in many cancers, including pancreatic cancer, a disease noted for its current lack of effective therapeutics. The K-Ras isoform of Ras is mutated in over 90% of pancreatic ductal adenocarcinomas (PDAC) and there is growing evidence linking aberrant PI3K/AKT/mTOR pathway activity to PDAC. Although these observations suggest that targeting one of these nodes might lead to more effective treatment options for patients with pancreatic and other cancers, the complex regulatory mechanisms and the number of sequence-conserved isoforms of these proteins have been viewed as significant barriers in drug development. Emerging insights into the allosteric regulatory mechanisms of these proteins suggest novel opportunities for development of selective allosteric inhibitors with fragment-based drug discovery (FBDD) helping make significant inroads. The fact that allosteric inhibitors of Ras and AKT are currently in pre-clinical development lends support to this approach. In this article, we will focus on the recent advances and merits of developing allosteric drugs targeting these two inter-related signaling pathways.

## Introduction

Dysregulation of cellular signaling pathways that involve phosphoryl transfer through GTPase and kinase activities is a key step in the initiation and development of neoplasms (Hanahan and Weinberg, 2011). Among the GTPases, constitutive Ras activation plays a critical role in the development of pancreatic, colon, and lung cancers, and is an indicator of poor prognosis (Kim et al., 2006; Califano et al., 2012; Pérez-Ruiz et al., 2012), with the Kristin isoform of Ras (K-Ras) being mutated in over 90% of pancreatic ductal adenocarcinomas (PDAC) (Morris et al., 2010). Until recently, development of therapeutically relevant K-Ras inhibitors had been unsuccessful due in large part to the protein's picomolar affinity for GDP and GTP (John et al., 1990). However, structural studies, including computational chemistry techniques (Grant et al., 2011) and fragment-based drug discovery (FBDD) (Maurer et al., 2012; Sun et al., 2012; Ostrem et al., 2013), have begun to explore novel allosteric-based opportunities for drugging this notoriously recalcitrant enzyme.

Phosphatidylinositol 4,5-bisphosphate 3-kinases (PI3K) form a key node in the PI3K/AKT/mTOR pathway, regulating numerous biological processes through phosphoryl transfer (Katso et al., 2001). Through its association with Ras, this family of kinases has been implicated as key driver in pancreatic cancer (Eser et al., 2013). Selectivity has been a major stumbling block in drugging PI3K since a large number of sequence-conserved isoforms play distinct yet overlapping functions within the cell (Vanhaesebroeck et al., 2010). However, as with Ras, computational chemistry techniques and FBDD have helped develop some promising new avenues in selectively targeting various isoforms (Giordanetto et al., 2011, 2012; Hughes et al., 2011), albeit through competitive inhibitors that bind the active site. Selective PI3K inhibition by allosteric means may soon emerge from recent reports that have begun to decipher the activation mechanism of these enzymes (Burke et al., 2012; Hon et al., 2012). Two other major nodes of the PI3K/AKT/mTOR pathway, AKT and mTOR, form additional focal points for the development of novel therapeutics. As with Ras and PI3K, these two enzymes have benefited from structural studies aimed at gaining greater understanding of their mechanisms of action and, as a consequence, have provided opportunity for novel allosteric-based approaches to their inhibition.

In the context of Ras and the PI3K/AKT/mTOR pathway, this mini-review discusses recent advances in the development of selective inhibitors with a focus on novel druggable allosteric sites, as well as highlighting some unexpected and undesirable outcomes associated with compounds that target catalytic sites. Some of the most salient areas of research are presented below and is intended to give the reader an overview of an exciting new arena of drug discovery.

## Inhibitors of Ras

Ras proteins form key nodes in a wide array of signaling pathways (Malumbres and Pellicer, 1998) and as a consequence aberrant Ras function can result in cancer. There are over 30 Ras-subfamily members (Wennerberg et al., 2005); due to their sequence similarity, number and diverse function, development of selective compounds is critical in minimizing the likelihood of drug toxicity. As shown below, the past few years have seen remarkable progress toward reaching this goal due in large part to the application of FBDD.

Structural studies have shown that Ras undergoes a large conformational change upon turnover, driven by hydrolysis of the γ-phosphate of GTP as seen from comparison of the GDP product-bound co-structure to a non-hydrolysable GTP analog (GMPPNP) co-structure (Milburn et al., 1990). In the GTP-bound “on” state, two sections of the active site, switch I and switch II, pack around the terminal phosphate. In the GDP-bound “off” state, with the γ-phosphate moiety removed, the catalytic site opens up, altering the surface topology. This impairs Ras' ability to activate a variety of effector proteins, including PI3K (Rodriguez-Viciana et al., 1994; Castellano and Downward, 2010). Mutations in switch I, switch II, or the p-loop (a region that recognizes the β-phosphate of the nucleotide) promote the oncogenic properties of Ras (Pai et al., 1989; Milburn et al., 1990; Prive et al., 1992; Scheffzek et al., 1997). Additionally, two classes of ancillary proteins directly interact and promote Ras' adoption of either the “on” or “off” state; GTPase-activating proteins (GAPs) enhance Ras' intrinsically slow GTPase activity to drive formation of the “off” conformer, and guanine nucleotide exchange factors (GEFs) promote exchange of GDP for GTP to switch the enzyme back to its “on” state.

Mutations in all four clinically relevant isoforms of Ras (H-Ras, N-Ras, and splice variants K-Ras-4A and K-Ras-4B) are known to cause a variety of cancers, with K-Ras being the most frequently mutated isoform in pancreatic cancer (COSMIC database, Sanger Institute). As mutations in Ras are common to a variety of cancers many different strategies that aim to down regulate constitutively active mutant forms have been explored (Gysin et al., 2011); however, until recently, lack of progress resulted in this family of proteins being largely regarded as undruggable (Sawyers, 2009). Development of competitive inhibitors that stabilize a GDP-bound inactive conformation must overcome Ras' picomolar affinity for GTP (John et al., 1990), inherently low GTPase activity (John et al., 1993), and high intracellular concentrations of GTP (Traut, 1994). Furthermore, the lack of any obvious druggable sites on the enzyme surface beyond the active site (Ahmadian et al., 1999) suggests inhibition is unlikely to be achieved by an allosteric approach. To circumvent these perceived barriers, compounds that inhibit farnesyl transferase, the enzyme that prenylates Ras at the C-terminus (Kohl et al., 1994; Lingham et al., 1998), were developed in an attempt to prevent Ras from co-localizing with effector proteins on the inner-face of the plasma membrane. Unfortunately, cells have been shown to maintain Ras prenylation, and therefore recruitment to the membrane, through gerenylgerenylation of the cysteine moiety (Whyte et al., 1997).

**Figure 1:**
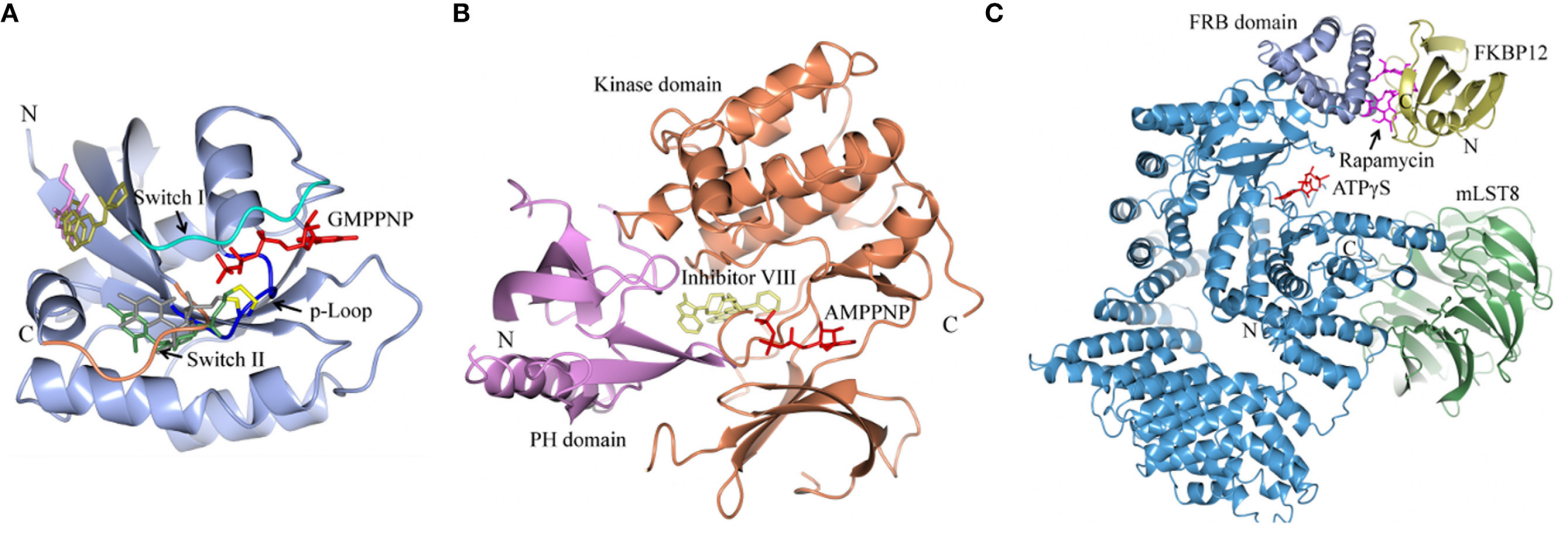
**Structures of human K-Ras, AKT1 and mTOR complexed with allosteric inhibitors**. For compound structures refer to Table 1, N- and C-terminal are highlighted. **(A)** The overall structure of K-Ras shown in ribbon representation (PDB ID: 3GFT) with switch I, switch II, and the p-loop highlighted. The active site is marked by nucleotide analog substrate, GMPPNP, and is illustrated in red. Allosteric inhibitors are shown with the following color scheme; 4,6-dichloro-2-methyl-3-aminoethyl-indole (PDB ID: 4DST), pink; compound 13 (PDB ID: 4EPY), gold; compound six (PDB ID: 4LUC), green; compound eight (PDB ID: 4LYF), gray. The latter two compounds are covalently attached to the side-chain of Cys13, highlighted by the Cβ and Sγ-atoms colored blue and yellow, respectively. **(B)** The overall fold of the PH and kinase domains of AKT1 shown in ribbon representation (PDB ID: 3O96), with the N-terminal PH colored pink and the C-terminal kinase domain in coral. The active site is marked out by AMPPNP (from PDB ID: 4EKK) and illustrated in red, inhibitor VIII is shown as pale yellow. **(C)** Crystal structure of mTOR^ΔN^, shown as blue ribbons, complexed with regulatory protein mLST8, shown as green ribbons (PDB ID: 4JSP). FKBP12 (PDB ID: 1FAP) (yellow) which recruits allosteric inhibitor rapamycin (shown in magenta) is superimposed onto the complex using mTOR's FRB domain (highlighted in slate blue) as a reference. Substrate analog ATPγS is shown in red.

New approaches to compound screening have begun to change the mindset of Ras being a poor drug target. Glycine 12 of K-Ras, which forms part of the p-loop, is a common locus for amino acid substitution in many cancers, with G12C being the fourth most frequently substituted amino acid in oncogenic K-Ras for both PDAC and cancers in general (COSMIC database, Sanger Institute). This observation prompted Ostrem and co-workers to use mass spectrometry to screen a library of fragment-like compounds that contain reactive sulfur moieties for ones which covalently modified K-Ras^G12C^ (an approach known as tethering) (Ostrem et al., 2013). Compounds were identified and, through medicinal chemistry efforts, lead compounds were developed that selectively modified mutant K-Ras by binding to a novel allosteric pocket, termed S-IIP, that is formed in part by the switch II motif (Figure 1A and Table 1B). Structural analyses of covalently modified protein demonstrated that Ras can be stabilized in a GDP-bound inactive conformation. Interestingly, two other groups have isolated compounds using FBDD that both bind K-Ras in a hydrophobic pocket adjacent to the switch I/II motifs (Figure 1A and Table 1B), blocking SOS-mediated nucleotide exchange (Maurer et al., 2012; Sun et al., 2012). Whether or not this new insight will aid in the development of non-covalent allosteric therapeutics that specifically target the more common G12V and G12D K-Ras amino acid substitutions that are associated with cancers of the pancreas and large intestine remains to be seen.

Table 1**(A) Allosteric inhibitors currently either FDA approved or in clinical trials that target major nodes of the PI3K/AKT/mTOR pathway. (B) A selection of compounds reported as allosteric inhibitors of Ras and major nodes of the PI3K/AKT/mTOR pathway**.

**Table:** 

**(A)**				
**Compound**	**Target**	**Cancer**	**Stage of development**	**Structure**
MK-2206	AKT1/2	Pancreatic/colon/breast/lung	Phase I/II	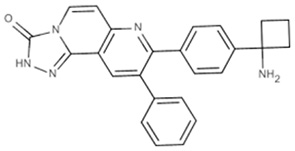
Everolimus	mTOR	Renal cell carcinoma	Approved	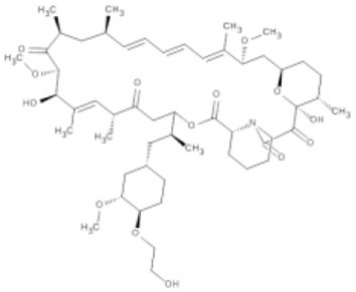
Temsirolimus	mTOR	Renal cell carcinoma	Approved	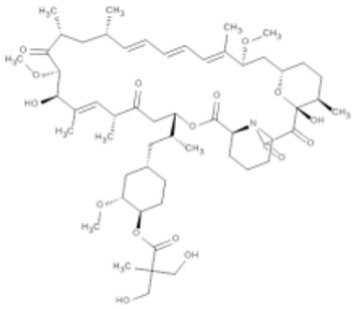

**Table:** 

**(B)**			
**Compound**	**Target**	**References**	**Structure**
Compound 6	K-Ras (G12C)	Ostrem et al., 2013	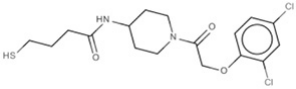
Compound 8	K-Ras (G12C)	Ostrem et al., 2013	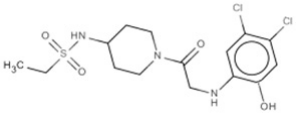
4,6-dichloro-2-methyl-3-aminoethyl-indole	K-Ras/ H-Ras	Maurer et al., 2012	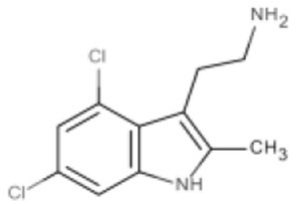
Compound 13	K-Ras	Sun et al., 2012	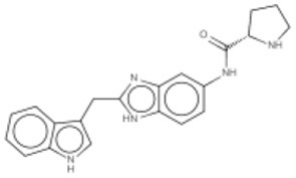
Inhibitor VIII	AKT1/2	Lindsley et al., 2005	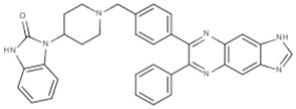

## Inhibitors of PI3K/AKT/mTOR pathway

The PI3K/AKT/mTOR pathway forms one of many mechanisms that regulate progression though the cell cycle and cellular apoptosis (Chang et al., 2003). Unsurprisingly, dysregulation of a component in this pathway can give rise to cancer. The three major nodes of the PI3K/AKT/mTOR pathway are: PI3K, a family of related kinases that primarily phosphorylate lipid-based phosphatidylinositol secondary messengers upon activation by receptors on the cell surface, AKT (Rac/protein kinase B), a kinase that binds the PIP (phosphatidylinositol phosphate) products of PI3K via its pleckstrin homology (PH) domain for recruitment to the plasma membrane, and mTOR (mammalian target of rapamycin). Due to the large and diverse array of down-stream effectors, each one of these three enzymes forms a node that has significant appeal in the development of therapeutics that target pancreatic and other cancers. Below we describe some of the efforts made toward this goal with an emphasis on the advantages gained by development of allosteric compounds.

### PI3K

The PI3K family is comprised of four classes of kinases that can be further divided based on their subunit assembly; they are composed of one of eight distinct catalytic subunits and, depending on the class, a regulatory subunit (Vanhaesebroeck et al., 2010) with p110α and p85α, being the most well characterized catalytic and regulatory subunits, respectively. The combination of catalytic and regulatory subunits defines individual PI3K function, with mutations in the corresponding genes associated with specific cancers (Liu et al., 2009).

Functional diversity necessitates the development of selective PI3K inhibitors. In contrast to other targets mentioned in this review, advances in PI3K selectivity have been brought about by the discovery and development of compounds that target the active site. Knight and colleagues made early insights into the development of selective non-allosteric inhibitors using *in vitro* assays to screen a panel of diverse PI3K inhibitors against PI3K family members (Knight et al., 2006). Three crystal structures of compounds bound to catalytic subunit p110γ were solved, including a p110δ-selective non-allosteric inhibitor that is a close analog of Idelalisib [previously known as GS-1101/CAL-101 (May et al., 2008)], a drug that has recently been approved by the FDA for the treatment of a number of blood-related cancers. From these structures, two regions lining the active site, an inducible hydrophobic “selectivity” pocket and an “affinity” pocket, were identified that are thought to contribute to the binding properties of a given ligand. It appears serendipity of target choice and the unique plasticity of the PIK3 active site rather than design have played the key roles in the development of selective PI3K competitive inhibitors as efforts to find further isoform-selective inhibitors using fragment-based screening methods have also produced compounds that target the active site (Giordanetto et al., 2011, 2012; Hughes et al., 2011).

Recent biophysical and structural data have begun to shed light on the allosteric mechanism of PI3K regulation. Burke and colleagues used results from mass spectrometry experiments with PI3K isoform p110α/p85α to propose an activation and recruitment model which occurs in four distinct steps in an as-of-yet unknown order (Burke et al., 2012): disruption of two distinct p110α/p85α interfaces (which are promoted by phosphorylated receptor tyrosine kinase and membrane binding), conformational change within the catalytic p110α subunit (possibly to allow correct orientation on the membrane surface), and lipid substrate binding at the active site. To further highlight the complexity of PI3K regulation, a recent report has shown through SPR and kinase activity assays that p110α substrate binding and kinase activity is modulated by membrane composition (Hon et al., 2012). Interestingly, this same group reported crystallographic evidence of a novel inducible ligand-binding site distinct from the active site of p110α, but were unable to demonstrate whether or not this could be exploited to inhibit kinase activity. Given that not all PI3K isoforms include a regulatory subunit, and that the activation mechanism of class I kinases is likely dependent on subunit composition (Burke et al., 2011; Zhang et al., 2011), additional selective PI3K inhibition though allostery might be achieved by taking advantage of multiple druggable sites rather than one site shared between all isoforms.

### AKT

AKT is an effector kinase downstream from PI3K forming another node of potential therapeutic value. There are three isoforms of AKT (AKT1-3) all containing an N-terminal PH domain that binds PIP3, a central AGC kinase domain, and a hydrophobic C-terminal domain. After recruitment to the plasma membrane through binding PIP3, AKT is activated by two phosphorylation events: one on its activation loop (Thr308, AKT1 numbering) by membrane-associated PDK1 and the other on the C-terminal hydrophobic motif by mTORC2 (Ser473) (Sarbassov et al., 2005). Mounting evidence suggests each isoform performs a specific function (Santi and Lee, 2010); however, most current knowledge on the importance of selectively targeting each isoform derives from their differing roles in the initiation and progression of cancers (Agarwal et al., 2013).

The PH and AGC kinase domains present attractive drug targets within AKT; small molecule binding to the AGC kinase domain would directly inhibit upregulated kinase activity, while binding to the PH domain would prevent recruitment to the plasma membrane and therefore hinder activation by PDK1. Both domains are highly conserved between the three isoforms [kinase domains are ~85% identical and the PH domains are ~60% identical (Barnett et al., 2005a)] as well as being conserved in a diverse array of functionally unrelated proteins, thus requiring development of compounds that exhibit a high degree of selectivity. There are four types of AKT inhibitors currently in development: ATP-competitive inhibitors, analogs of PIP3, substrate peptidomimetics, and allosteric modulators (Kumar and Madison, 2005; Collins, 2009). ATP-competitive inhibitors are the most mature in terms of development, with the first series of AKT selective compounds reported in 2002 based on a PKA inhibitor (Reuveni et al., 2002); however, significant obstacles remain due to the isoforms possessing near identical nucleotide-binding sites (Kumar and Madison, 2005) and that competitive inhibitors have been found to unexpectedly hyperphosphorylate AKT through an intrinsic regulatory mechanism (Okuzumi et al., 2009).

A decade ago Merck reported on the discovery of compounds that only inhibit AKT kinase activity in the presence of the PH domain (Barnett et al., 2005b). Enzyme kinetics on full length and PH domain deleted AKT constructs showed these compounds to be allosteric inhibitors. Subsequent work by the team resulted in the development of MK-2206 (Table 1A), a drug that shows moderate selectivity for AKT1/2 over AKT3 (Yan, 2009), and is currently in clinical trials for a wide variety of cancers. The structure of another isoform-selective allosteric inhibitor produced by this research program, inhibitor VIII [(Lindsley et al., 2005) and Table 1B], has been solved in complex with an AKT1 construct containing both PH and kinase domains, giving structural evidence of an allosteric regulatory mechanism in which changes in relative positioning of the two domains regulates activity (Wu et al., 2010). The inhibitor was shown to bind AKT1 between the PH and kinase domains (Figure 1B) to lock the protein in a “PH-in” conformation, preventing ATP access to the nucleotide-binding site. Furthermore, the authors inferred that active site competitive inhibitors hyperphosphorylate AKT as a consequence of only binding to a “PH-out” conformation, preventing the PH domain from fully closing onto the kinase domain. This would result in keeping the phospholipid-binding site exposed and enhancing AKT's recruitment to the membrane where it becomes phosphorylated. These observations suggest how allosteric approaches to drugging a target might offer significant advantages over more conventional approaches to enzyme inhibition.

### mTOR

Mammalian target of rapamycin (mTOR) is a member of the serine/threonine phosphatidyl inositol 3′ kinase-related kinase family (PIKK) that uniquely phosphorylates protein substrates and is sensitive to rapamycin, a large macrocyclic compound that is synthesized by *Streptomyces hygroscopicus* as an antifungal agent (Vezina et al., 1975). Rapamycin does not directly bind mTOR alone, but forms a complex with cyclophilin FKB12. This protein-inhibitor complex inhibits kinase activity by binding to mTOR's FKBP12-rapamycin binding (FRB) domain and restricting access to the active site (Huang et al., 2003; Yang et al., 2013). mTOR is part of two multi-protein complexes, mTORC1, composed of mTOR, mLST8, DEPTOR, RAPTOR, and AKT1S1/PRAS40, and mTORC2, composed of mTOR, mLST8, DEPTOR, PRR5, RICTOR, and MAPKAP1. mTORC2 is regarded as lacking sensitivity to rapamycin due to RICTOR preventing mTOR-FKBP12-rapamycin complex formation (Sarbassov et al., 2004); however, prolonged exposure appears to overcome this through inhibition of free mTOR prior to its assembly into the mTORC2 complex (Sarbassov et al., 2006). Both complexes regulate cell growth, but perform distinct roles within the cell; mTORC1 promotes mRNA translation, ribosome biogenesis, and autophagy, while mTORC2 promotes entry into the cell cycle, cell survival, remodeling of the cytoskeleton, and metabolism (Sabatini, 2006). As part of mTORC1, mTOR serves as a downstream effector of AKT, where activated AKT indirectly activates mTORC1 (Inoki et al., 2003). In contrast, mTORC2 phosphorylates AKT at Ser473 to upregulate pathway activity (Sarbassov et al., 2005). Surprisingly, rapamycin-based inhibition of mTORC2 has been shown to paradoxically increase levels of AKT phosphorylation; however, in contrast to the intrinsic mechanism of AKT hyperphosphorylation by AKT competitive inhibitors, this has been shown to occur through an extrinsic negative feedback mechanism that results in Ser473 hyperphosphorylation by PI3K (O'Reilly et al., 2006). This observation suggests that a combinatorial therapy that attenuates feedback loops, such as addition of a PI3K inhibitor, might be an effective way to increase drug efficacy.

Being a member of the PIKK family, it is unsurprising that many compounds developed to target the ATP-binding site of PI3K can also inhibit mTOR (Garcia-Echeverria and Sellers, 2008). In contrast, rapalogs (analogs of rapamycin) are selective allosteric inhibitors of mTOR, with Temsirolimus and Everolimus being approved by FDA for the treatment of renal cell carcinomas. The recent crystal structure of a truncated mTOR that includes the FAT (focal adhesion targeting), FRB (FKBP12-rapamycin-binding) and kinase domains sheds some light on the mechanism of its regulation (Yang et al., 2013); using the FRB domain as a point of reference, the FKBP12 subunit can be superimposed onto the complex and suggests the FKBP12-rapamycin complex inhibits mTOR activity by occluding access to the active site (illustrated in Figure 1C). The authors mapped hyperactivating mutational hotspots onto the structure to show clustering at three sites and rationalized that the majority of these mutations have a destabilizing effect, particularly on an inhibitory α-helix that results in improved access to the active site. Such effects on protein structure could present opportunities in the development of allosteric compounds that stabilize an inactive state, as that shown for K-Ras.

To the best of the authors' knowledge, there are no publications applying methods such as FBDD to develop non-rapamycin-based compounds that target selective allosteric sites remote from the active site or to interfere with mTORC1/mTORC2 assembly. Additionally, all known mTOR selective drugs currently in clinical trials cannot distinguish between mTORC1 and mTORC2. Given the size of the protein (290 kDa) and the complex nature of its multi-protein assembly, there is likely significant opportunity to develop selective drugs that modulate mTORC1/mTORC2 activities and hence deliver more fine-tuned treatments for cancer.

## Future developments

Allosteric compounds are making it into mainstream drug development due in part to recent advances in structural biology and biophysical techniques, FBDD, and computational chemistry. As summarized in this mini-review, recent drug discovery programs have shown that allosteric inhibitors may have an edge over more common active site-competitive inhibitors, particularly when targeting specific members of a family of related proteins. An added benefit to this approach appears to be the reduction in likelihood of obtaining unexpected and undesired results like that seen in hyperphosphorylated AKT. The emerging knowledge of allosteric regulatory mechanisms of therapeutically important targets, such as PI3K, may aid in future allosteric drug development, although caution should be noted, as some targets are likely to remain intractable to allosteric inhibition by their intrinsic nature.

Unlike the discovery and development of drugs that target active sites, allosteric drug discovery may present more of a challenge as methodologies for detection and analysis of hit and lead compounds are still maturing. However, emerging technologies, such as micro-scale thermophoresis for compound screening and diversity oriented synthesis for library design, may help accelerate the discovery and development of drugs that allow effective treatment of currently incurable cancers such as PDAC.

### Conflict of interest statement

The authors declare that the research was conducted in the absence of any commercial or financial relationships that could be construed as a potential conflict of interest.
